# Wastewater inflow time series forecasting at low temporal resolution using SARIMA model: a case study in South Australia

**DOI:** 10.1007/s11356-022-20777-y

**Published:** 2022-05-20

**Authors:** Phuong Do, Christopher W. K. Chow, Raufdeen Rameezdeen, Nima Gorjian

**Affiliations:** 1 Sustainable Infrastructure and Resource Management (SIRM), UniSA STEM, University of South Australia, Mawson Lakes, Adelaide, SA 5095 Australia; 2 Future Industries Institute, University of South Australia, Adelaide, SA 5095 Australia; 3 South Australian Water Corporation, Adelaide, South Australia Australia

**Keywords:** Wastewater inflow, Forecasting, Time series modelling, SARIMA model

## Abstract

Forecasts of wastewater inflow are considered as a significant component to support the development of a real-time control (RTC) system for a wastewater pumping network and to achieve optimal operations. This paper aims to investigate patterns of the wastewater inflow behaviour and develop a seasonal autoregressive integrated moving average (SARIMA) forecasting model at low temporal resolution (hourly) for a short-term period of 7 days for a real network in South Australia, the Murray Bridge wastewater network/wastewater treatment plant (WWTP). Historical wastewater inflow data collected for a 32-month period (May 2016 to December 2018) was pre-processed (transformed into an hourly dataset) and then separated into two parts for training (80%) and testing (20%). Results reveal that there is seasonality presence in the wastewater inflow time series data, as it is heavily dependent on time of the day and day of the week. Besides, the SARIMA (1,0,3)(2,1,2)_24_ was found as the best model to predict wastewater inflow and its forecasting accuracy was determined based on the evaluation criteria including the root mean square error (RMSE = 5.508), the mean absolute value percent error (MAPE = 20.78%) and the coefficient of determination (*R*^2^ = 0.773). From the results, this model can provide wastewater operators curial information that supports decision making more effectively for their daily tasks on operating their systems in real-time.

## Introduction

The operations of wastewater pumping systems or networks consume a tremendous amount of electrical energy to transfer sewage and with both the financial and energy inefficiency issues which can be handled by improving management practices (Galve et al. 2021; Mirra et al. 2020). Meanwhile, practical guidance for pumping operations is generally not available; thus, wastewater operators activate or deactivate the pumps only according to their own expert knowledge and experience of the system generally resulting in higher operating energy costs (Kim et al. 2006). A pump switching program that properly controls pump on/off applied into the wastewater network can lead to a great reduction in energy costs (Wei et al. 2013), especially when the pumps are planned to operate with the precise estimation of electricity spot market prices and wastewater inflow rates (Do et al. 2022, 2021). Wastewater inflow forecasting plays an essential role in controlling pumping system of a wastewater network (Piri et al. 2021). The quantity of incoming wastewater to the network/wastewater treatment plant (WWTP) can be used to pre-schedule pump operations. Therefore, to achieve optimal schedules for wastewater pumps, it is best to forecast influent flow rate in advance as one of the significant parameters (Kim et al. 2016; Zeng et al. 2016; Wei and Kusiak 2015).

In the recent literature, there have been studies focused on projections of wastewater inflow rate to the WWTPs using different data-driven approaches which can be separated into three categories. The first one is the machine learning (ML) method. Wei et al. (2013) applied four ML algorithms, including multilayer perceptron neural network (MLPNN), random forest, boosted tree, and support vector machine to model the quantity of influent flow. The MLPNN was determined as the best-performing algorithm and therefore chosen to produce forecasts. In addition, other ML techniques were also used to predict wastewater inflow rate such as chaos neural network (Li et al. 2007), k-nearest neighbour (Kim et al. 2016) and deep learning (Oliveira et al. 2020). The second data-driven method is the hybrid technique such as adaptive neural fuzzy interference–grey wolf optimiser (ANFIS-GWO) (Dehghani et al. 2019) and multimodal and ensemble-based deep learning (ME-DeepL) (Heo et al. 2021). The last one is the conventional data-driven method such as the autoregressive integrated moving average (ARIMA) model. ARIMA is developed with a time series which is a set of data acquired at evenly spaced time intervals; therefore, it is also called time series model. It has been proven as an effective method in constructing forecasting models for wastewater inflow to WWTPs. Kim et al. (2006) anticipated daily influent rate and properties by developing an ARIMA model based on daily data collected for 150 days. Research outcomes showed good forecast results for 1–7 days ahead. Nevertheless, to enhance the reliability of the proposed forecasting model, a sufficient data quantity was required as the collected datasets did not exhibit seasonal and annual patterns. ARIMA models were also able to describe weekly (Abunama and Othman 2017) and daily (Boyd et al. 2019) observed and future behaviour of wastewater inflow rate to produce forecasts for case study WWTPs. A comparison study was implemented by Zhang et al. (2019) on forecasting ability of the ARIMA and MLPNN models. The ARIMA model was developed using the wastewater inflow data only, while the MLPNN model included exogenous meteorological variables (e.g. temperature, precipitation). The results indicated reliable daily predictions could be obtained by both models. However, the ARIMA model was proven to have higher accuracy in terms of statistical metrics.

Predicting wastewater flow into the WWTPs is a challenging task. According to Zhang et al. (2019), engineers and operators have to cope with a number of uncertainties and complexities, such as the difficulties in simulating influencing factors on wastewater inflow (e.g. rainfall, runoff and infiltration) and the changes in infrastructure due to aging conditions. Time series models such as ARIMA and its derivatives (e.g. seasonal autoregressive integrated moving average (SARIMA), a model is formed by adding seasonal terms to the ARIMA model to deal with seasonal elements in the data series) can overcome these problems (Zhang et al. 2019). The dynamics of the wastewater inflow rate is expected to follow a certain pattern such as time of the day, day of the week, weekly, monthly or quarterly which means there is a presence of seasonality in the time series. ARIMA model is inadequate for forecasting in this case; therefore, a seasonal ARIMA (SARIMA) approach needs to be applied to develop predictive models (Hyndman and Athanasopoulos 2018) to address the shortcoming of the ARIMA method.

SARIMA technique has been used to build forecasting models in a wide range of scientific disciplines such as hydrology, meteorology, and climatology (Brito et al. 2021; Liu et al. 2021; Ray et al. 2021). However, there is no comprehensive evaluation of the ability, and reported application of the SARIMA model to forecast wastewater inflow has been found. Besides, in the studies of the ARIMA model as mentioned above, researchers only explored predictive ability of this model for high temporal resolution including daily and weekly forecasts. In a real-time control system, wastewater inflow used as smart controller’s input to plan pump on/off schedules in advance should be predicted for low temporal resolution. From these knowledge gaps arise the need for further research on the development of a wastewater inflow forecasting model with temporal resolution of 60 min to generate hourly forecasts for pumping control in real-time. Moreover, this need leads to the framing of a research argument on the forecasting performance of the SARIMA model for hourly wastewater inflow.

This study describes the application of the SARIMA model as a predicting approach to address the seasonality in the wastewater inflow time series and forecast future datapoints. The primary purposes of this paper are to characterize wastewater inflow rate and develop a SARIMA model as an inflow forecasting tool for the Murray Bridge WWTP. The accuracy of the proposed model was evaluated based on three statistical indexes, including the root mean square error (RMSE), the mean absolute value percent error (MAPE) and the coefficient of determination (*R*^2^). The main objectives of this study are as follows: (1) identifying and selecting the best SARIMA forecasting model for a real wastewater network/WWTP, (2) generating low temporal resolution (60 min) wastewater inflow forecasts for a short-term period (7 days) and (3) managerial implications regarding the application of hourly inflow predictions in the real-time wastewater pumping control.

This paper proceeds as follows. The methodology describes the methods used for modelling and forecasting low temporal resolution (hourly) wastewater inflow, the research case study, the process of collecting and preparing data, the step-by-step procedure of forecasting model development and criteria to evaluate its accuracy. The findings on wastewater inflow pattern investigation, model development and prediction are provided as results and discussed. Finally, the conclusion gives a summary and highlights the outcomes of the study.

## Methodology

### SARIMA model

ARIMA time series model (Box et al. 2015) relies on the analysis of historical data to predict future values with an assumption that data patterns in the past can be utilized to predict data in the future. The ARIMA model consists of three components, including (i) autoregression (AR) which describes the correlation between an observation with its own lagged values, (ii) integration (I) which shows the number of times differencing needs to be performed to make the data series stationary, and (iii) moving average (MA) which represents the correlation between observations and residual errors (Wang et al. 2021; Parmezan et al. 2019).

SARIMA is developed by including additional seasonal component to the ARIMA model which handles the seasonality in the time series. SARIMA model, in general, is a combination of the non-seasonal module (p,d,q) and seasonal module (P,D,Q)_s_ with seven parameters. It is denoted as SARIMA(p, d, q)(P, D, Q)_s_ (1); where *p* and *P* are the order of non-seasonal and seasonal AR term; *d* and *D* are the degree of non-seasonal and seasonal differencing; *q* and *Q* are the order of non-seasonal and seasonal MA term; and *s* is the length of seasonality in the time series. For example, in an hourly time series, *s* = 24; in a daily time series, *s* = 7; in a monthly time series, *s* = 12; and in a quarterly time series, *s* = 4.

SARIMA, as a member of the ARIMA model family, works best when it is applied for a long and stable time series (Dimri et al. 2020). SARIMA method requires a medium to long length time series that consists of at least 50 data points. It has a strong dependence on the historical data; therefore, the continuity of data is required to be guaranteed (Zhou et al. 2019).

The ARIMA model family such as AR and ARIMA has been a widely used technique for wastewater inflow predictions. However, the SARIMA model, an extended version of the ARIMA model has not yet been applied in the same filed. The ARIMA model is utilized if there is no presence of seasonality in a time series. In case a seasonality pattern exists, the SARIMA model needs to be applied (Hyndman and Athanasopoulos 2018). In the time series data, seasonality is observed when the changes in data have a regular pattern that repeats over a certain period. Seasonality is a known and fixed frequency cycle (Hyndman and Athanasopoulos 2018). There are different seasonality types such as time of the day, day of the week, weekly, monthly and quarterly.

## Case study

**Fig. 1:**
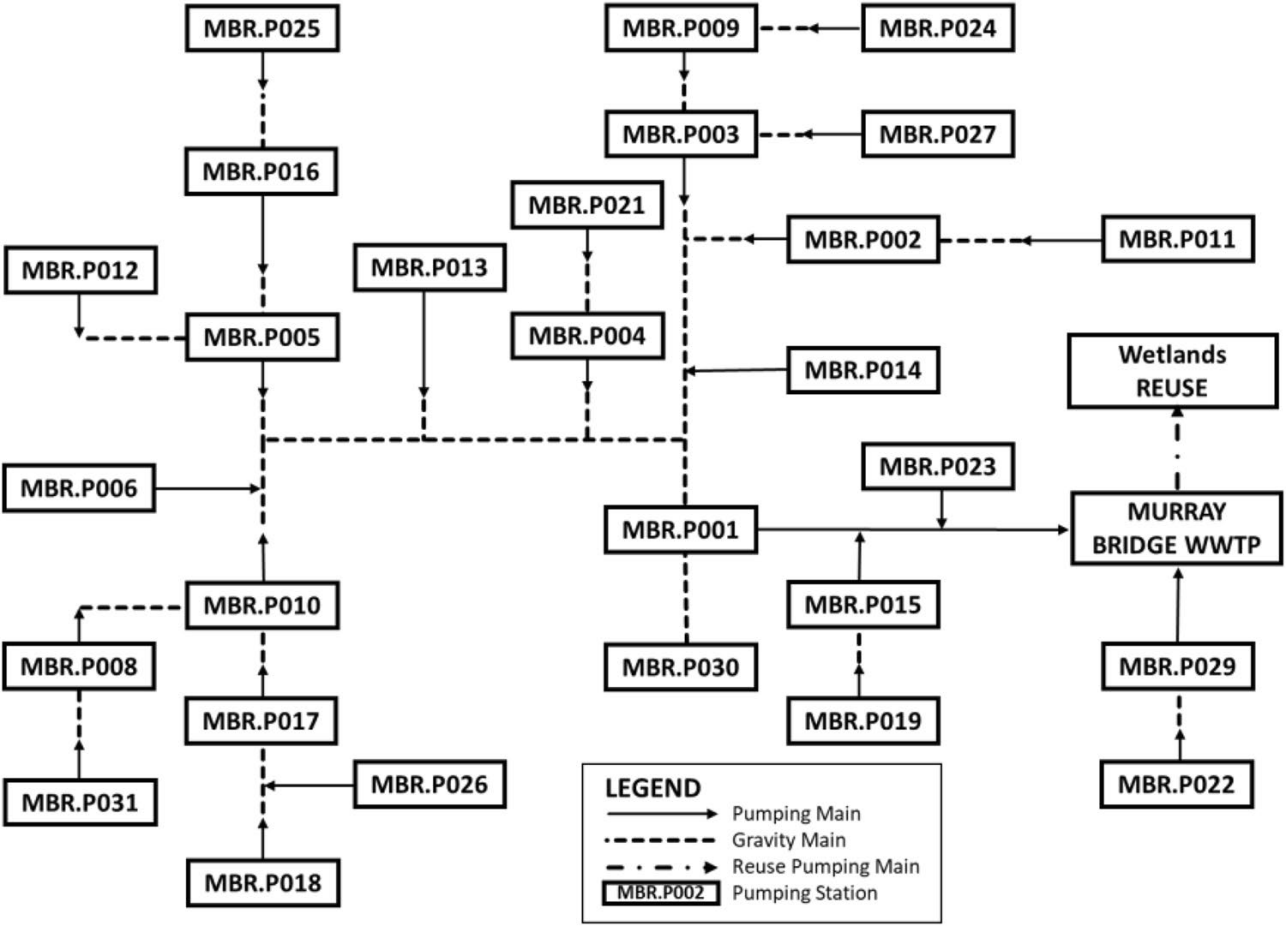
Schematic diagram of the Murray Bridge wastewater network

The Murray Bridge wastewater network in South Australia, a realistic wastewater network with real data was selected as the case study to apply the proposed SARIMA model. It serves approximately 14,000 people and covers an area of about 14 km^2^ with different land-use types (e.g. residential, commercial, education and recreational). Details of this network and related studies have been published in Do et al. (2021), Gorjian Jolfaei et al. (2019) and Konetschka et al. (2017). Figure 1 shows the schematic diagram of this case study.

In this study, an assumption has been made that the total wastewater inflow to the Murray Bridge WWTP is considered to be equal to the total flow collected from sources/catchments then transferred by numerous pump stations in the Murray Bridge wastewater network. At the WWTP, the flow meter is installed; therefore, data is available to be used to develop the forecasting model for wastewater inflow.

### Data collection and pre-processing

**Fig. 2:**
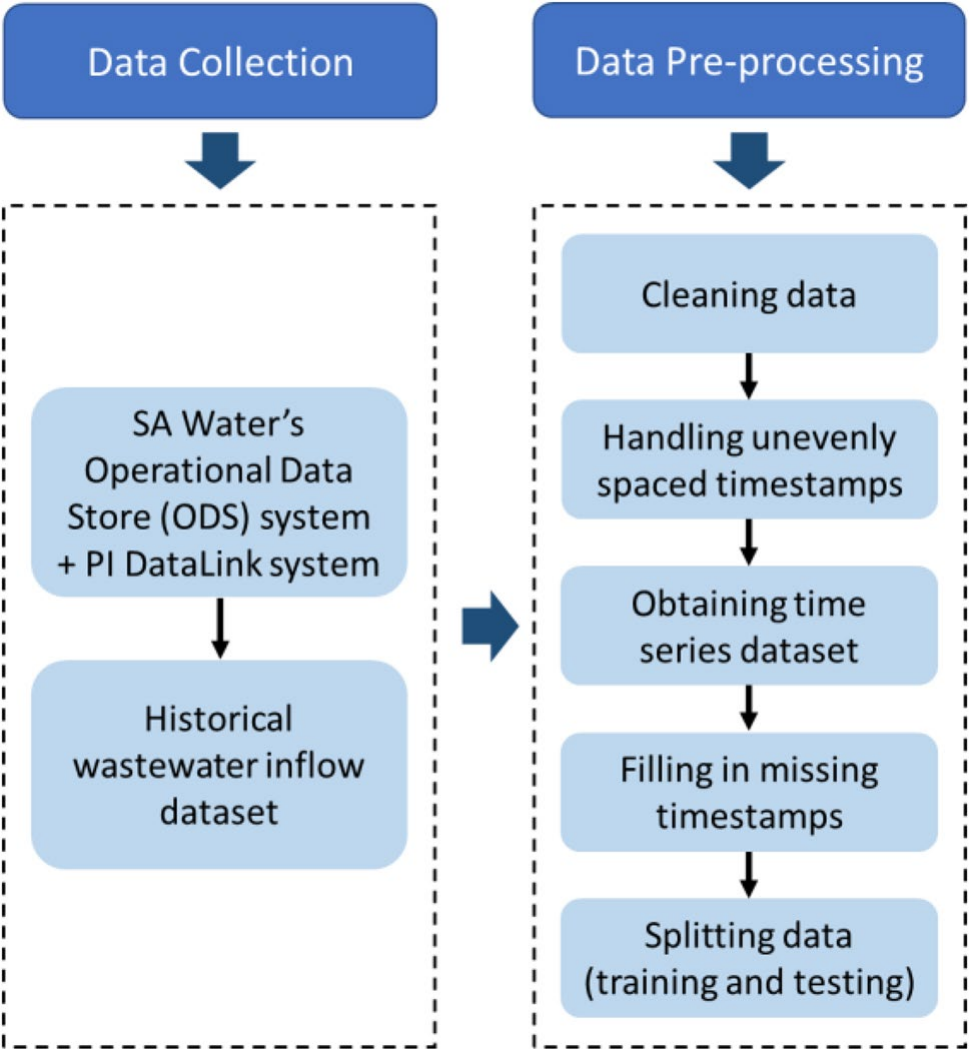
Data collection and pre-processing procedures

Data preparation was conducted with two stages: (1) collection and (2) pre-processing to gather and transform raw data into a time series dataset used for modelling and forecasting wastewater inflow. The procedures are described in Fig. 2.

- Data collection
  The historical wastewater inflow data of the Murray Bridge WWTP for 32 months from 7 May 2016 to 31 December 2018 were sourced from the SA Water’s Operational Data Store (ODS). The raw dataset with 1,149,637 data points in total was unevenly spaced which included actual measurements of inflow rate to the WWTP at different sampling times. They were recorded at intervals of 3 s at a minimum and 3 h 50 min at a maximum, and mostly every 5 and 55 s.
- Data pre-processing

Data cleaning was first conducted to identify and handle probable inaccurate or irrelevant data. There were 504 data errors detected, including 484 non-numerical, 16 abnormally large and four negative values. They were determined by sorting the dataset ascending and descending. All of them were eliminated to achieve a more consistent and better accuracy dataset to build a predictive model for wastewater inflow.

The filtered wastewater inflow dataset with 1,149,133 data points remaining after error elimination was converted to an hourly time series dataset by averaging data within each 60 min. After the data conversion process, each day in the considered period has 24 records; therefore, an hourly wastewater inflow dataset with 23,256 data points was created. This converted dataset was then inspected to find out any interval without data. Twenty detected missing values accounting for only 0.09% of the entire converted dataset were filled in by averaging the two nearby observations which are close to the average value of this dataset; therefore, there was no impact on the data.

The hourly wastewater inflow dataset was divided into two parts: training and testing. As stated by Hyndman and Athanasopoulos (2018), typically, the size of the testing set accounts for around 20% of the entire dataset and is ideally at least equal to the longest forecasting duration. Therefore, the ratio of training to testing set is 80:20. The training set that includes data of the first 26 months (May 2016 to June 2018) with 18,840 data points was used for model development. The testing set that consists of data of the remaining 6 months (July to December 2018) with 4416 data points was reserved for model validation. A summary of details of the wastewater inflow datasets is shown in Table 1.

**Table 1:** Wastewater inflow dataset summary

Dataset		Data points	Starting date	Ending date
Raw data		1,149,637	7 May 2016	31 December 2018
Filtered data		1,149,133	7 May 2016	31 December 2018
Converted data (hourly data)	Entire set	23,256	7 May 2016	31 December 2018
	Training set	18,840	7 May 2016	30 June 2018
	Testing set	4416	1 July 2018	31 December 2018

### Model development procedure

**Fig. 3:**
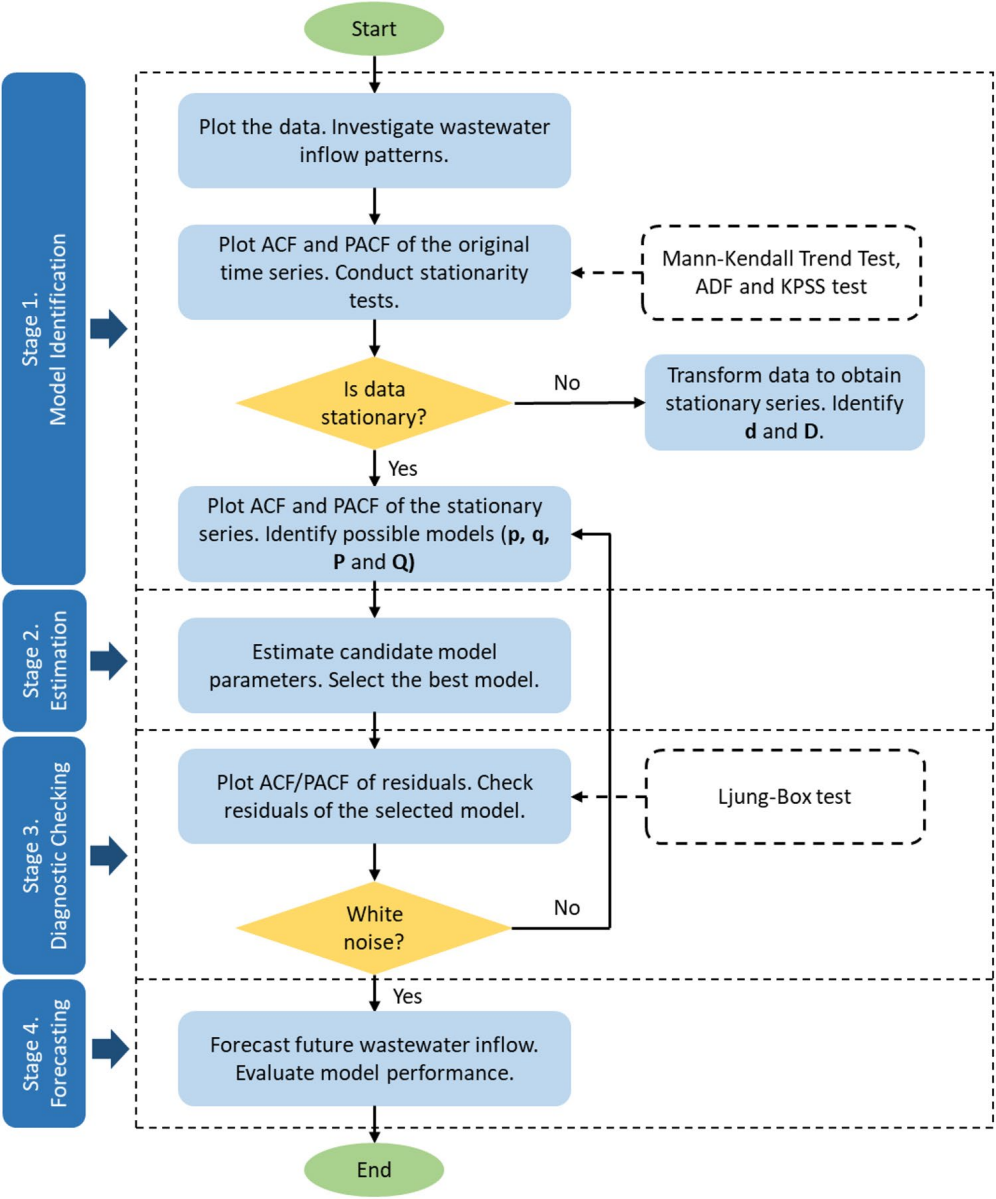
Flowchart of modelling and forecasting wastewater inflow procedure

Figure 3 illustrates the flowchart of the step-by-step methodology applied for modelling and forecasting wastewater inflow in this study. The procedures were based on Box and Jenkins methodology (Box et al. 2015) and comprised four stages, including (i) model identification, (ii) parameter estimation, (iii) diagnostic checking and (iv) forecasting. IBM SPSS Statistics 25 was employed as a tool to support the implementation of these four stages.

The procedures of these four stages are described in detail as follows.

- Stage 1. Model identification

The first and most important requirement for the development of SARIMA model is to ensure the wastewater inflow rate time series data is stationary. A time series is considered to be stationary when its statistical features (e.g. mean and variance) are constant over time, or not impacted by time at which the series is observed. The term “stationarity” is used to imply the stationary status of a time series. In contrast, when a time series exhibits trends (e.g. upward or downward) and/or seasonal patterns (e.g. quarterly, monthly or weekly), it is non-stationary.

On the basis of the above-mentioned requirement, the first step of the model identification stage was checking the stationarity of the data. The training set (see Table 1) was used in this stage and from this section onwards, it is called the original training time series. This wastewater inflow dataset was plotted to provide an initial guess about its stationarity features (Jalil and Rao 2019). Boxplots of wastewater inflow grouped by time of the day and day of the week were used to analyse possible intraday and intraweek patterns visually. Next, to statistically test the data stationarity, the Mann–Kendall Trend Test (Kendall 1975; Mann 1945) was undertaken to examine whether there is an increasing or decreasing trend in the time series. This is a commonly used test for hydro-meteorology time series such as streamflow, rainfall and temperature (Kabbilawsh et al. 2020). The Mann–Kendall test is a non-parametric test that is less impacted by the presence of outliers compared to other parametric tests (Praveen et al. 2020; Wang et al. 2020; Hamed 2009). Additionally, further statistical tests were also implemented to mathematically confirm the stationarity condition of the training wastewater inflow series, including a unit root test, Augmented Dickey-Fuller (ADF) test (Dickey and Fuller 1979) and a stationarity test, Kwiatkowski-Philips-Schmidt-Shin (KPSS) (Kwiatkowski et al. 1992). These three tests were performed at the 5% significance level where the alpha value was 0.05 (*α* = 0.05) corresponding to the 95% confidence interval. Two opposing hypotheses were set up for each test, including null hypothesis H_0_ and alternative hypothesis H_a_. The purpose of hypothesis tests was to decide between H_0_ and H_a_ with rules applied for rejecting the null hypothesis H_0_. Table 2 summarizes statements of the null and alternative hypotheses and decision rules applied for the trend, unit root and stationarity tests.

**Table 2:** Summary of hypothesis testing for stationarity check

Test	Null hypothesis H_0_ and alternative hypothesis H_a_	Decision rules
Mann–Kendall test	H_0_: There is no trend in the series H_a_: There is a trend in the series	If *p*-value ≤ *α* = 0.05, H_0_ is rejected If *p*-value > *α* = 0.05, H_0_ is failed to be rejected
ADF	H_0_: There is a unit root for the series. The series is non-stationary H_a_: There is no unit root for the series. The series is stationary	
KPSS	H_0_: The series is stationary H_a_: The series is non-stationary	

After checking the stationarity of the training time series using statistical tests, the non-seasonal differencing *d* and seasonal differencing *D* were determined. If the series is stationary, it is not required to execute the process of differencing, and the value of parameters *d* and *D* is zero. In case the series is non-stationary with the presence of seasonality and trend, the seasonal difference is applied. When there is no trend and seasonality component, the series is transformed by the non-seasonal difference. The value of parameters *d* and *D* implies the number of times the wastewater inflow series needs to be differenced to satisfy stationarity. The autocorrelation function (ACF) and partial autocorrelation function (PACF) plots of the original training time series are created if required to further confirm its stationarity. In this study, the ACF plots depict the correlation coefficient between the wastewater inflow time series and its own lagged values, and the PACF plots measure the partial correlation coefficient between this data series and lagged versions of itself.

The next step was to plot the ACF and PACF of the stationary time series. It could be the original training time series with stationary status or the differenced series after differencing process obtained from the previous step. The non-seasonal and seasonal orders of AR (parameters *p* and *P*) and MA (parameters *q* and *Q*) were identified based on the ACF and PACF plots. Different values of those parameters were combined to identify possible configurations of (p,d,q) and (P,D,Q) for potential SARIMA models.

- Stage 2. Parameter estimation
  In this stage, various potential models identified in stage 1 were examined. The coefficient of determination (*R*^2^), root mean square error (RMSE), and normalized Bayesian information criterion (BIC) were used to select one amongst the potential models. The best model with the optimal set of parameters has the highest *R*^2^, and the least RMSE and normalized BIC.
- Stage 3. Diagnostic checking
  The best model selected in stage 2 was tested to determine whether it adequately captured the behaviour of the wastewater inflow data to the Murray Bridge WWTP. The correlograms ACF and PACF of residuals were plotted to check if the residuals followed a white noise process after fitting a SARIMA(p,d,q)(P,D,Q)_s_ model to the time series. The difference between observed and fitted data is called residuals. The residuals are white noise when they are identically, independently distributed with a zero mean. If at least 95% of all lags lie within the lower and upper confidence limits, it can be concluded that the selected model can be used for the analysis of the wastewater inflow series.
  The Ljung-Box Test (Ljung and Box 1978) was also conducted to detect white noise in the residual time series. The hypotheses used for the Ljung-Box test include a null hypothesis H_0_ that means the residuals are white noise, and an alternative hypothesis H_a_ that means the residuals are not white noise. They were performed at the 5% significance level (*α* = 0.05). If *p*-value ≤ *α* = 0.05, H_0_ is rejected, while if *p*-value > *α* = 0.05, H_0_ failed to be rejected, and H_a_ is accepted.
- Stage 4. Forecasting

A model with the highest accuracy in simulating wastewater inflow would be employed to forecast data. Applying the selected SARIMA model, the wastewater inflow series were forecasted using the SPSS software. The predicted values were then matched against the testing set.

### Model performance evaluation

In order to determine the precision of the SARIMA model in wastewater inflow predictions, the root mean square error (RMSE), the mean absolute value percent error (MAPE) and the coefficient of determination (*R*^2^) were used as statistical indicators to evaluate the fit of the forecasted to the observed values. Lower values of RMSE and MAPE and a higher value of *R*^2^ imply a more reliable and robust model (Ansari et al. 2018).

## Results and discussion

### Visualization of the data patterns

**Fig. 4:**
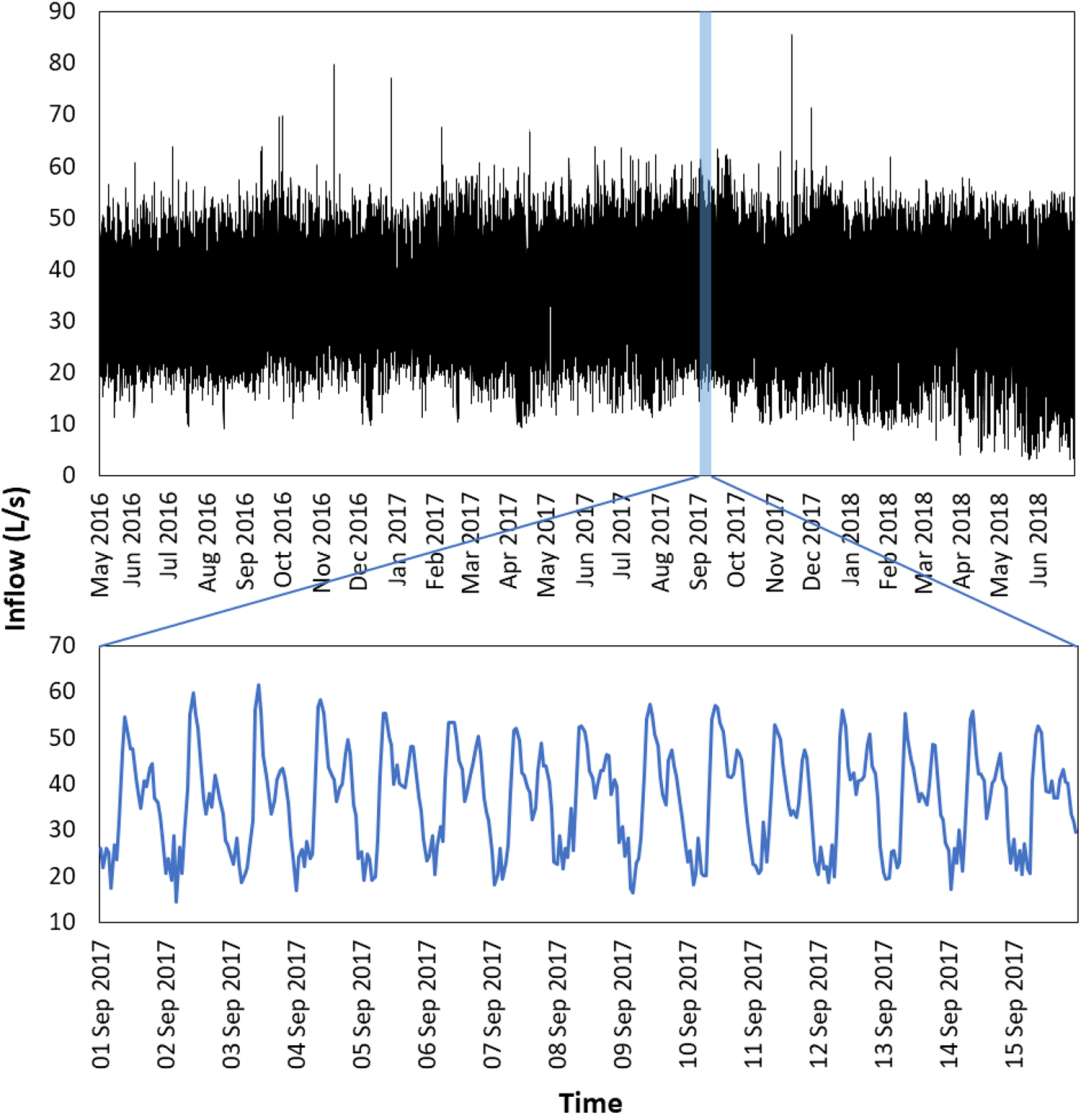
Hourly wastewater inflow to the Murray Bridge WWTP time series plot of the training set (7 May 2016–30 June 2018) and a random zoom (1–15 September 2017)

The original training time series was used as input for the process of modelling and forecasting hourly wastewater inflow to the WWTP. A plot of this dataset (May 2016 to June 2018) is generated as in Fig. 4. Using this plot, trend and seasonality of the series could be visually identified. A random zoom for the 1–15 September 2017 period was provided to achieve a better insight into the dynamics of the hourly wastewater inflow rates. From this zoom, it could be preliminarily determined that the wastewater inflow rate had no trend and tended to be very low from the beginning of each day, then reached a peak twice during the day. This indicates possible intraday patterns in the time series. Further investigation on trend and seasonal of the series in terms of time of the day and day of the week will be presented in the next section.

### Wastewater inflow patterns

**Fig. 5:**
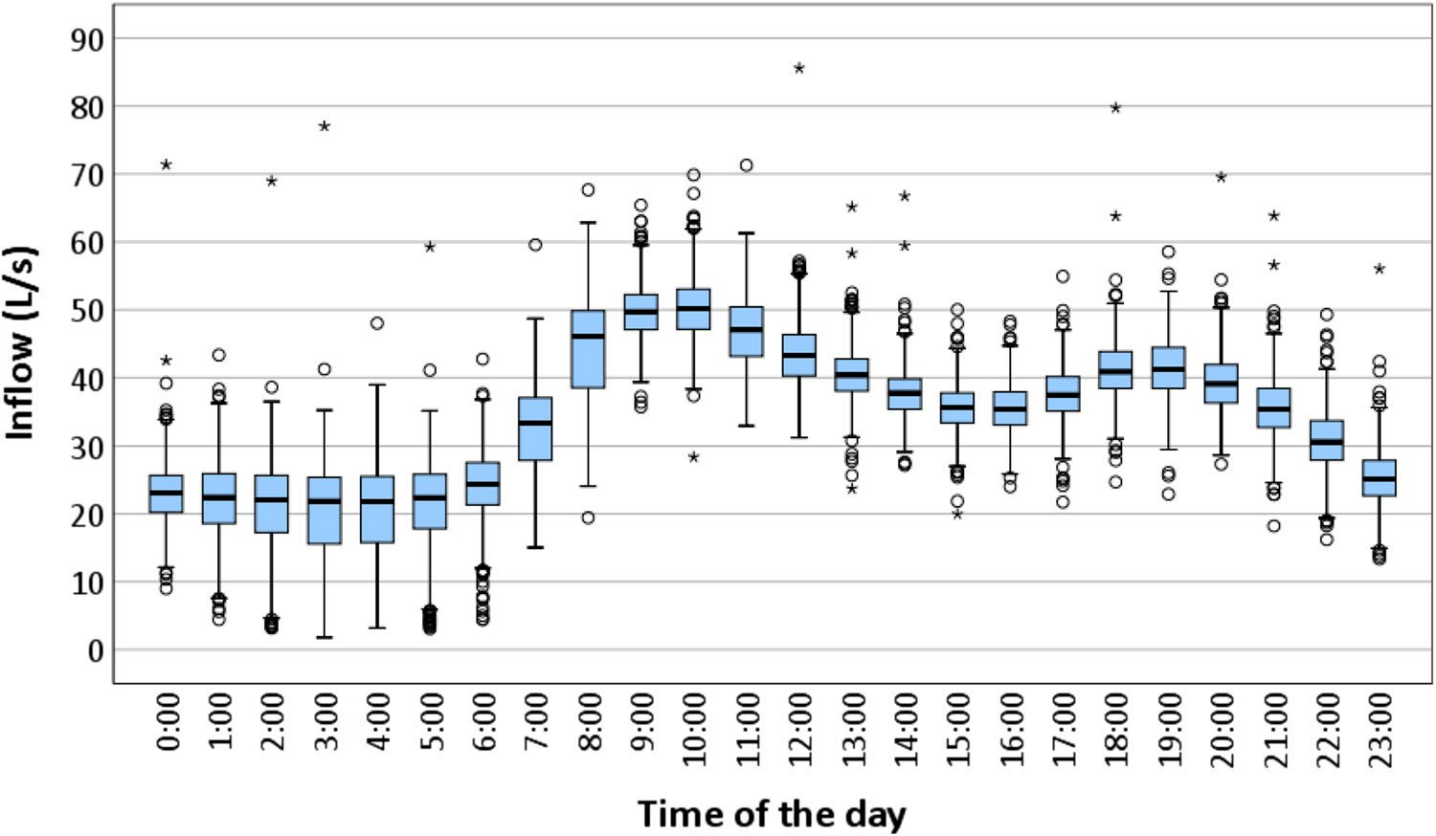
Wastewater inflow pattern by time of the day (7 May 2016–31 December 2018)

Time of the day patterns of the wastewater inflow to the Murray Bridge WWTP are revealed in Fig. 5 in the form of boxplots. The inflow rates were low after midnight till 5:00. The higher wastewater inflow occurred in the early morning, late afternoon and early evening. In particular, from 6:00, it increased then peaked at 10:00. It can be said that hours during the day have a strong influence on the daily high and low wastewater inflow rates. This implies the existence of the intraday seasonality in the wastewater inflow dataset.

**Fig. 6:**
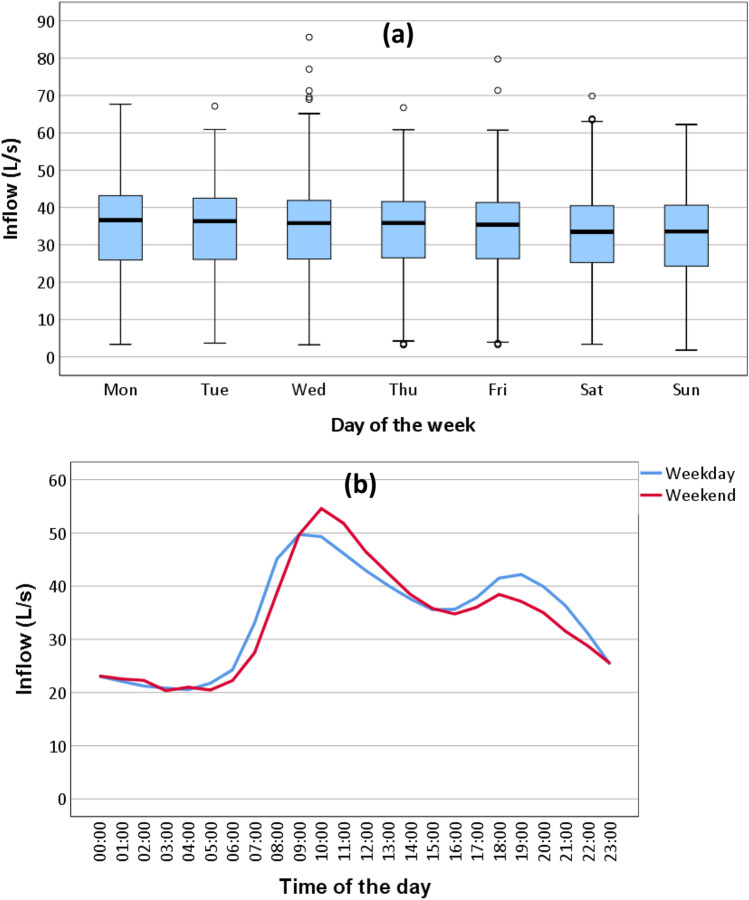
(**a**) Wastewater inflow patterns by day of the week and (**b**) hourly average wastewater inflow by time of the day and by weekday and weekend (7 May 2016–31 December 2018)

Boxplots grouped by day of the week of wastewater inflow data are shown in Fig. 6a. Mondays were often the days with the highest inflow to the network/WWTP which was just slightly greater than that of other weekdays (Tuesdays to Fridays). The inflow rate on Saturdays and Sundays was slightly lower than the remainders of the week. The lower rate on weekends compared to weekdays by time of the day is also shown in Fig. 6b. At every hour of the day, excluding 6 h from 09:00 to 15:00, the weekday inflow rate was higher than that of the weekend. The difference between the higher and lower levels of wastewater inflow by weekdays and weekends indicates it is dependent on the day of the week. Therefore, there is a presence of intraweek seasonality in the wastewater inflow data series.

With the identified intraday and intraweek seasonality components, it can be stated that the wastewater inflow series is non-stationary. To statistically claim the presence of stationarity in the time series, several trend and stationarity tests were required to implement.

### Data stationarity tests

Stationary is a compulsory condition of the data time series to be used for a SARIMA model. If the series is still non-stationary even after certain times of differencing, it is failed to apply the model (Zhang et al. 2019). Before employing the SARIMA technique to develop a forecasting model, the time series data needs to be in a stationary condition. Therefore, the stationarity of the original training time series of hourly wastewater inflow was investigated. The Mann–Kendall trend test, the ADF and KPSS tests, and the ACF and PACF plots were used to verify the data’s stationarity. The results of these statistical tests can be seen in Table 3.

**Table 3:** Results of the stationarity tests of the original training data series

Parameter	Mann–Kendall	ADF	KPSS
*p*-value	0.160*	< 0.0001**	< 0.0001**
*α*	0.05	0.05	0.05
Reject null hypothesis H_0_	No	Yes	Yes
Stationary series	Yes	Yes	No

*One-tailed; **two-tailed.

For the Mann–Kendall Trend Test, the calculated *p*-value (0.16) was greater than the significance level *α* = 0.05 indicating that the null hypothesis H_0_ failed to be rejected. The result shows there is no downward or upward trend, and the time series is stationary. The ADF test showed the same outcome as the Mann–Kendall trend test. With the *p*-value of < 0.0001 lower than 0.05, the non-stationary null hypothesis was rejected. This confirms there is no unit root in the wastewater inflow series; therefore, the series is stationary. However, the KPSS test indicated a contrary outcome to the other two tests. The calculated *p*-value (< 0.001) was smaller than the significance level *α* = 0.05. Thus, the null hypothesis H_0_ was rejected which means the wastewater inflow series is concluded to be non-stationary. This may be caused by the strong seasonality of the series as analysed in the previous sections.

**Fig. 7:**
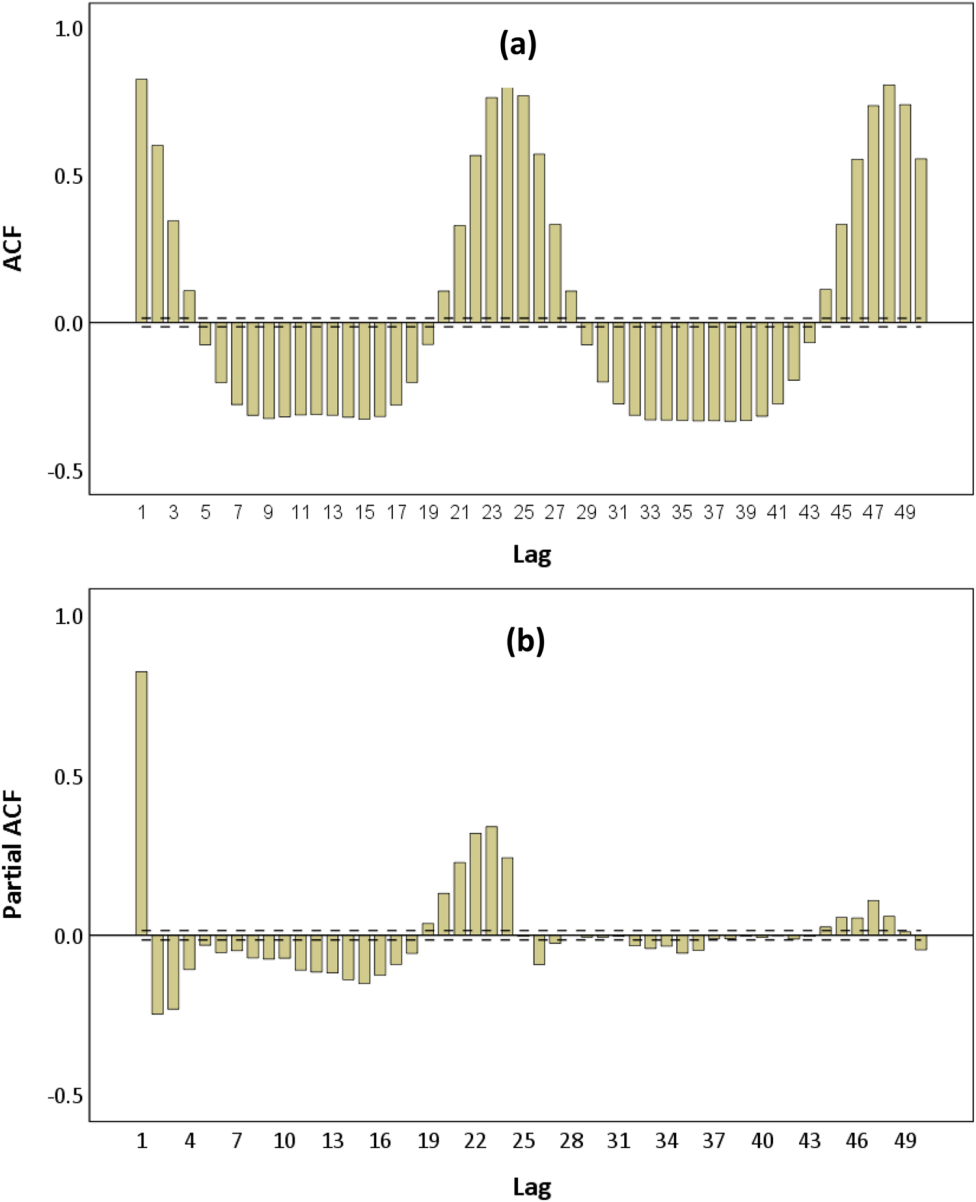
(**a**) ACF and (**b**) PACF plots of the original training wastewater inflow time series

The disagreement between results of the KPSS and other tests can be solved by examining the ACF and PACF coefficients of the original training time series (Kabbilawsh, Sathish Kumar & Chithra 2020). They are calculated by SPSS and plotted as in Fig. 7a and b. The black dashed lines in each ACF and PACF plot represent the 95% confidence level. The first 50 lags were analysed.

**Fig. 8:**
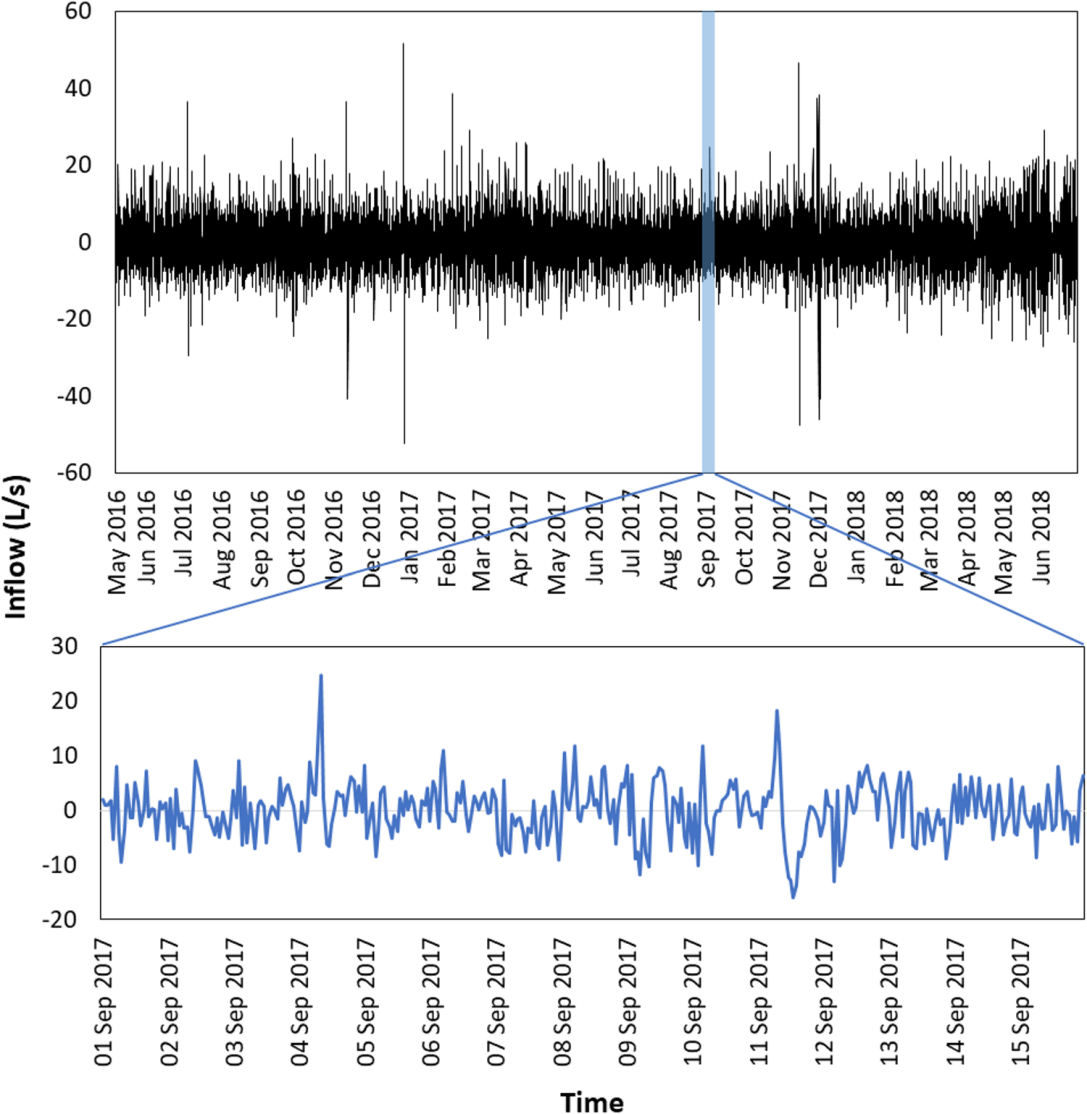
Plot of the first order seasonal differenced hourly wastewater inflow for the training time series (d = 0, D = 1, and s = 24) and a random zoom (1–15 September 2017)

From the ACF plot (Fig. 7a), there are significant peaks at 24 lags such as lag 24 and lag 48 which shows a strong seasonality repeating every 24 time points or 24 h in a day. It can be said that the wastewater inflow data is seasonal with period of seasonality *s* = 24. The ACF coefficients move in a sinusoidal wave pattern that is clear evidence of the presence of seasonality that makes the original training time series non-stationary. The existence of seasonality (or seasonal components) in a time series can be subtracted by seasonal differencing technique (Mills 2019; Brockwell and Davis 2016) . Therefore, the first order seasonal differencing *D* = 1 and periodicity *s* = 24 were performed to convert the original training time series to the stationarity form and satisfy the requirement of SARIMA modelling. Figure 8 shows the transformed hourly wastewater inflow training time series and a zoom for the first 15 days of September 2017. The fluctuations of this series at zero and constant mean demonstrate that it is stationary.

The first order seasonal differenced series obtained by the transformation process was then verified for stationarity using three statistical tests, including the Mann–Kendall Trend Test, ADF and KPSS tests. Table 4 reports results of these three stationarity tests for the first order seasonal differenced series. The *p*-value resulting from the Mann–Kendall and KPSS test was greater than *α* = 0.05 which means it was failed to reject the corresponding null hypotheses. For the ADF test, the null hypothesis was rejected. All these results infer that the first order seasonal differenced series is stationary and can be used for the SARIMA application.

**Table 4:** Stationarity tests for the first order seasonal differenced series

Parameter	Mann–Kendall	ADF	KPSS
*p*-value	0.968*	< 0.0001**	1.000**
*α*	0.05	0.05	0.05
Reject null hypothesis H_0_	No	Yes	No
Stationary series	Yes	Yes	Yes

*One-tailed; **two-tailed.

During the process of analysing and converting the non-stationary wastewater inflow series into stationary, the non-seasonal differencing was not required, so the value of parameter *d* is zero. With the seasonal difference *D* = 1 and period of seasonality *s* = 24 as identified previously, SARIMA(p,0,q)(P,1,Q)_24_ models were suggested for further investigation. In the next section, values of parameters *p*, *q*, *P* and *Q* will be found.

### Model selection

**Fig. 9:**
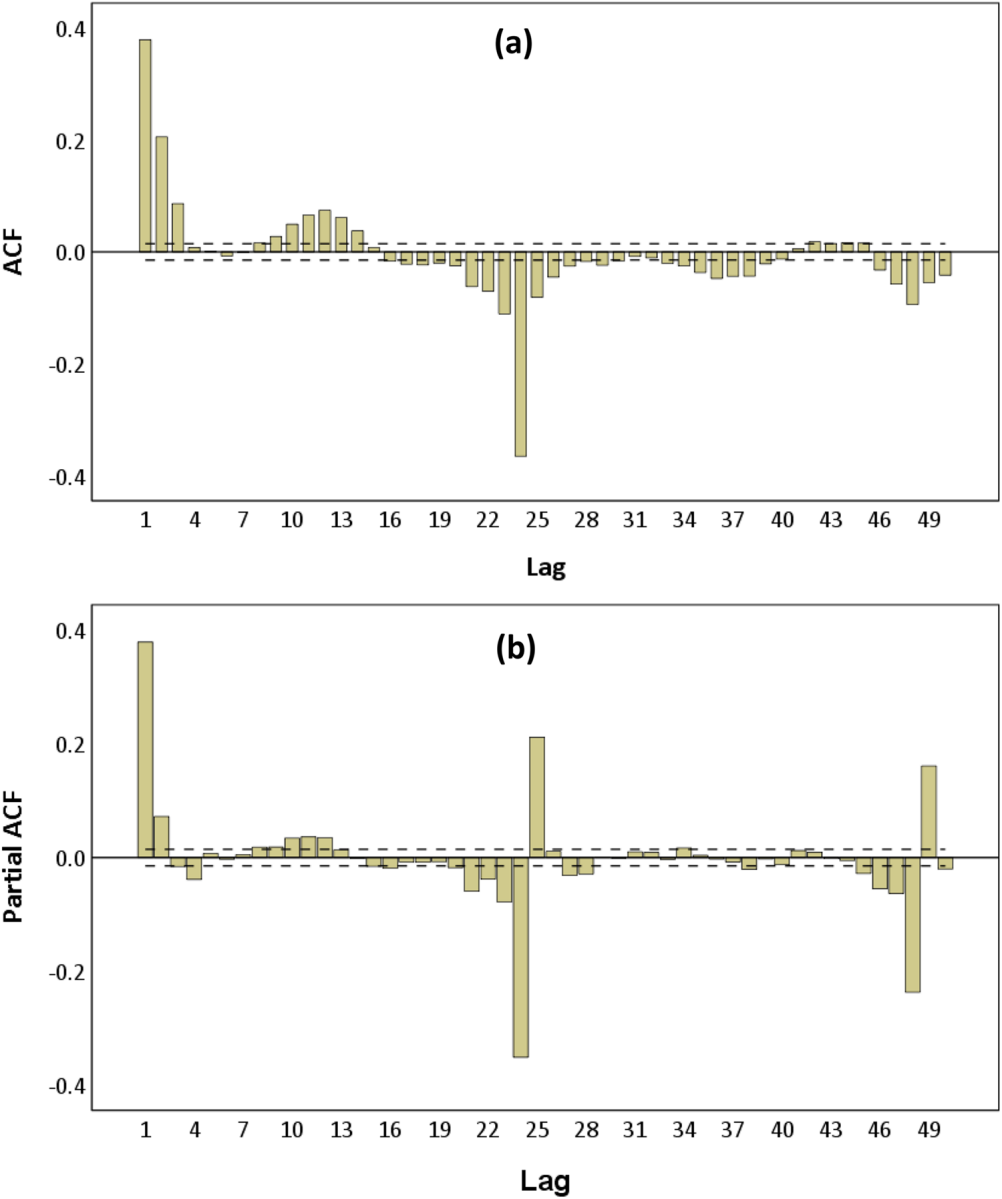
(**a**) ACF and (**b**) PACF plots of the first seasonally differenced series (d = 0, D = 1 and s = 24)

The SARIMA(p,0,q)(P,1,Q)_24_ model were ascertained by potential values for the non-seasonal AR order (*p*), non-seasonal MA order (*q*), seasonal AR order (*P*), and seasonal MA order (*Q*). ACF and PACF plots of the stationary wastewater inflow series which was seasonally differenced with *D* = 1 and periodicity *s* = 24 (see Fig. 9a and b) were used to identify the unknown parameters.

The behaviour of seasonal lags (e.g. lags 24 and 48) and non-seasonal lags which are lags of the first span of periodicity (lags 1–23) in the ACF plot (Fig. 9a) was investigated to determine the parameters *q* and *Q*, while in the PACF plot (Fig. 9b), the parameters *p* and *P*. From the ACF plot, at lags 1, 2 and 3, significant autocorrelations crossed outside the lower and upper confidence limits that indicate appropriate values of the parameter *q*. The continuity of significant autocorrelations disappeared at lags 4–7 as they lied between the lower and upper confidence limits. Thus, the significant autocorrelations at other non-seasonal lags in the first seasonal multiples of 24 were not taken into consideration. There were also significant autocorrelations at lags 12 and 24 of the ACF plot. This means the parameter *Q* could be 1 or 2. Similarly, significant autocorrelations at lags 1 and 2 observed from the PACF plot imply the potential values of parameter *p*. Seasonal lags 12 and 24 in the PACF plot with significant autocorrelations indicate 1 and 2 could be the values of the parameter *P*. With possible values of *p*, *P*, *q* and *Q*, 24 configurations of those parameters were combined corresponding to 24 potential models.

### Determine the optimum parameters

The selection of the best fitting model from 24 potential ones was based on the lowest RMSE and normalized BIC and the highest *R*^2^. Table 5 presents the results of those evaluation metrics for all potential models.

**Table 5:** SARIMA potential models

	SARIMA model	RMSE	Normalized BIC	*R*^2^
1	(1,0,1)(1,1,1)_24_	4.127	2.838	0.848
2	(1,0,2)(1,1,1)_24_	4.124	2.837	0.849
3	(1,0,3)(1,1,1)_24_	4.122	2.836	0.849
4	(2,0,1)(1,1,1)_24_	4.126	2.838	0.849
5	(2,0,2)(1,1,1)_24_	4.120	2.835	0.849
6	(2,0,3)(1,1,1)_24_	4.122	2.837	0.849
7	(1,0,1)(1,1,2)_24_	4.125	2.837	0.849
8	(1,0,2)(1,1,2)_24_	4.122	2.836	0.849
9	(1,0,3)(1,1,2)_24_	4.120	2.836	0.849
10	(2,0,1)(1,1,2)_24_	4.123	2.837	0.849
11	(2,0,2)(1,1,2)_24_	4.118	2.835	0.849
12	(2,0,3)(1,1,2)_24_	4.120	2.836	0.849
13	(1,0,1)(2,1,1)_24_	4.123	2.836	0.849
14	(1,0,2)(2,1,1)_24_	4.120	2.835	0.849
15	(1,0,3)(2,1,1)_24_	4.118	2.835	0.849
16	(2,0,1)(2,1,1)_24_	4.122	2.836	0.849
17	(2,0,2)(2,1,1)_24_	4.116	2.834	0.849
18	(2,0,3)(2,1,1)_24_	4.118	2.835	0.849
19	(1,0,1)(2,1,2)_24_	4.118	2.834	0.849
20	(1,0,2)(2,1,2)_24_	4.115	2.833	0.849
21	(1,0,3)(2,1,2)_24_	4.113	2.833	0.850
22	(2,0,1)(2,1,2)_24_	4.116	2.834	0.849
23	(2,0,2)(2,1,2)_24_	4.113	2.833	0.849
24	(2,0,3)(2,1,2)_24_	4.131	2.842	0.848

Both SARIMA(2,0,2)(2,1,2)_24_ and SARIMA(1,0,3)(2,1,2)_24_ had the smallest values of RMSE (4.113) and normalized BIC (2.833). However, the value of *R*^2^ for SARIMA(1,0,3)(2,1,2)_24_ was higher than that of SARIMA(2,0,2)(2,1,2)_24_ and the highest amongst other models (0.850). It can be concluded that SARIMA(1,0,3)(2,1,2)_24_ is the best model which satisfies the given conditions.

### Diagnostic checking

**Fig. 10:**
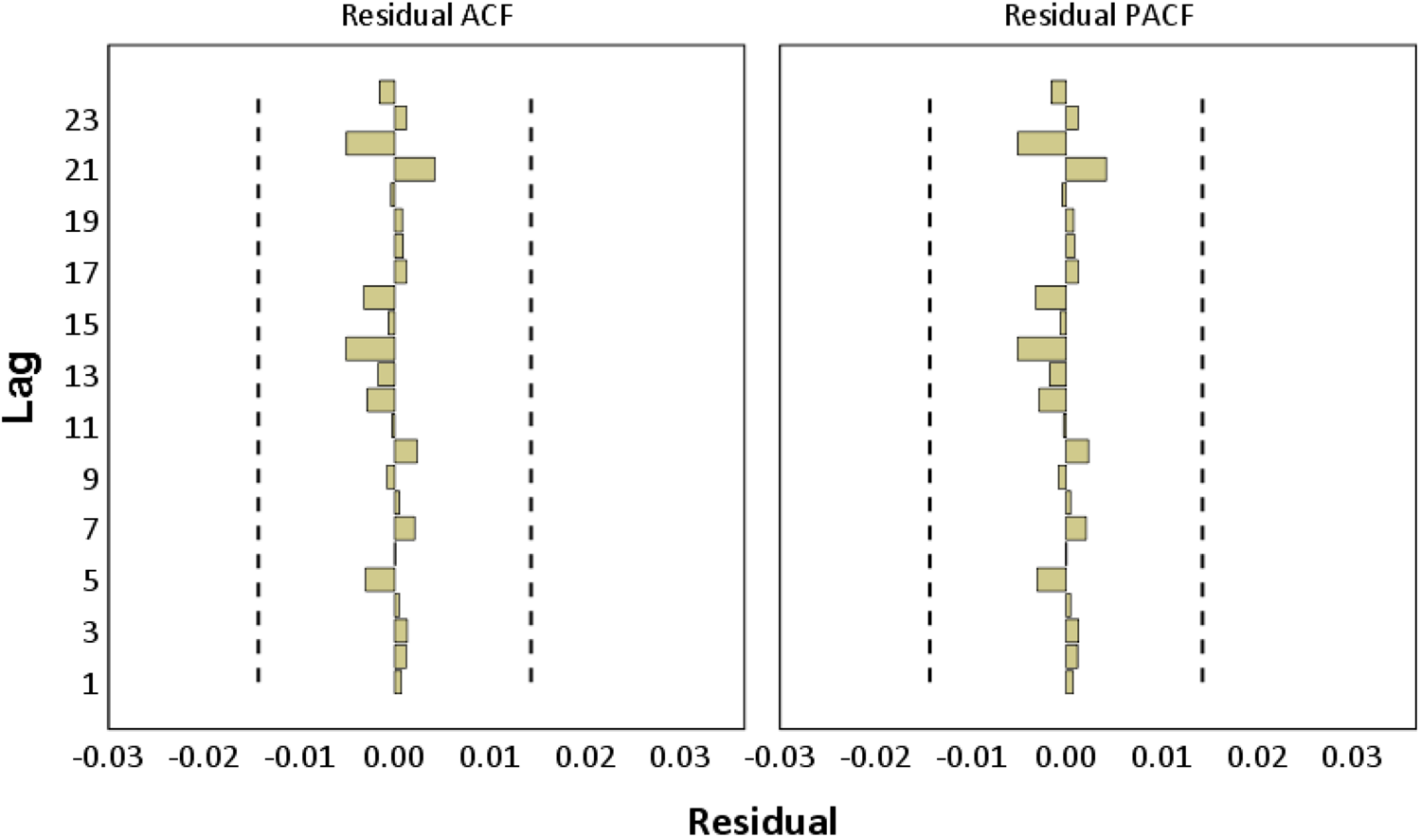
Residuals of ACF and PACF of the SARIMA(1,0,3)(2,1,2)24 model

Diagnostic checking was conducted with the purpose of testing the residuals of the best model SARIMA(1,0,3)(2,1,2)_24_ to identify if the SARIMA model sufficiently represents the statistical features of the observed wastewater inflow time series. Figure 10 shows the ACF and PACF residual plots of the selected model. All residuals lie between 95% confidential limits that indicate there is no autocorrelation amongst residuals; thus, the residuals are white noise.

White noise of the residuals was further tested by the Ljung-Box test to mathematically confirm its absence or existence. This is a diagnostic tool to determine if the residuals of a time series model are independent and identically distributed or if autocorrelation in a time series is different from zero. The results showed that the *p*-value (0.19) was greater than 0.05 indicating the null hypothesis failed to be rejected. This means the residuals are white noise. In other words, SARIMA(1,0,3)(2,1,2)_24_ removes the residual dependency from the wastewater inflow time series. Therefore, the proposed model passes the required check.

### Wastewater inflow forecasting

The ability of the proposed SARIMA model in predicting wastewater inflow data was assessed in this last stage. The testing dataset (1 July to 31 December 2018) was used for the model validation procedure. The SARIMA(1,0,3)(2,1,2)_24_ model was directly utilized for the entire testing process. There were no forecasts generated outside the testing period, as this study mainly focuses on the demonstration of the ability of the developed model in predicting future values rather than the actual wastewater inflow rate predictions for the case study WWTP.

The fitness of the observed and forecasted hourly wastewater inflow rate is discussed to determine the quality of the proposed SARIMA model. The mean of the observed and forecasted wastewater inflow rate was 33.26 L/s and 33.35 L/s, respectively. The difference between these two values is only 0.03% that indicates a good fit relationship. In additional, the results of statistical tests were as follows: RMSE = 5.508, MAPE = 20.78% and *R*^2^ = 0.773. The RMSE about two times lower than the standard deviation (11.56) is an indication of the good prediction (Boyd. et al. 2019). A high-quality forecasting model also has a low value of MAPE. In the previous studies, the results of MAPE were within the range of 71–78% in Zhang et al. (2019) and from 20 to 94% for 4 out of 5 case study WWTPs in Boyd et al. (2019). It can be concluded that a low MAPE value was achieved in this research. Besides, the value of *R*^2^ which is larger than 0.5 also indicate relatively good predictions (Alsharif et al. 2019). As a result, SARIMA(1,0,3)(2,1,2)_24_ is considered as a reliable forecasting model for wastewater inflow to the Murray Bridge wastewater network/WWTP.

**Fig. 11:**
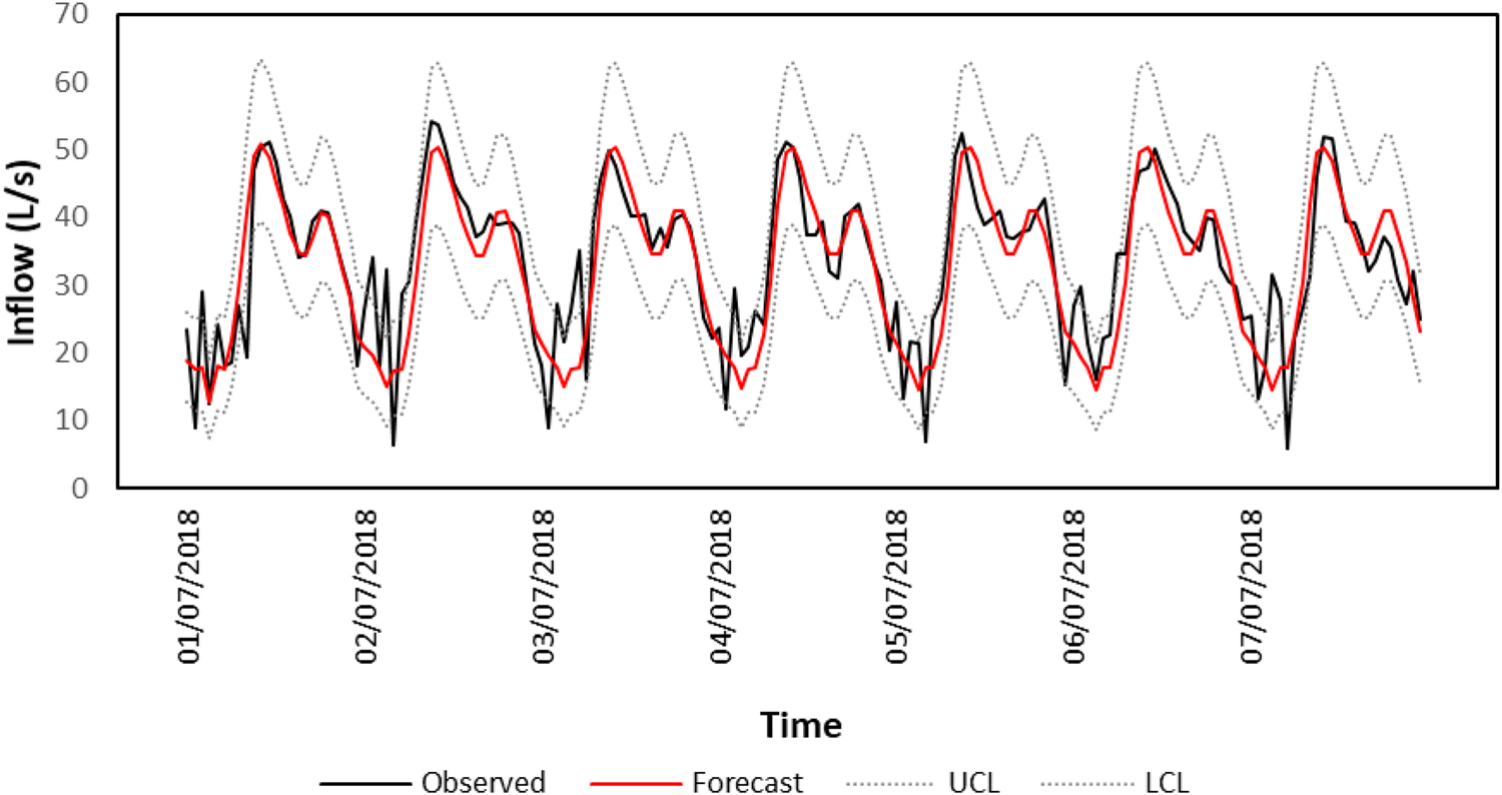
Observed and forecast wastewater inflow from 1 to 7 July 2018

Future forecasts data for the 1-week period (1–7 July 2018) using the proposed SARIMA model are illustrated in Fig. 11. The figure also compares observed and forecasted wastewater inflow, and 95% upper confidential limit (UCL) and lower confidential limit (LCL). SARIMA(1,0,3)(2,1,2)_24_ in general has the capability to provide future predictions for the wastewater inflow. The forecasted data relatively matched the observed data during morning until midnight from 6 a.m. to 12 a.m. However, it underestimated/overestimated the wastewater inflow during after midnight hours from 1–5 p.m. This could be because ARIMA family models only approximate the data patterns in the past, as the structure of the underlying data mechanism is not explained (YoosefDoost et al. 2017).

The predictions of wastewater inflow to the Murray Bridge wastewater network/WWTP generated based on an hourly time series dataset are useful for the smart wastewater pump controller. This controller operates pumps with consideration of two inputs including wastewater inflow rate and electricity spot price. Details on this smart controller are presented in Do et al. (2021). The forecasts of its two inputs can support the operators in planning the pump schedules during the day in advance. Therefore, wastewater inflow forecasts for low temporal resolution such as 60 min as in this paper are required to accomplish the task. It is impossible with daily and weekly predictions as in previous studies of ARIMA model. According to Dehghani et al. (2019), 7–10 days ahead forecasts offer sufficient time to schedule pumps. Thus, the forecasts every hour for a short-term period of 1 week ahead can significantly contribute to preparing operations plans for the WWTP.

## Conclusions

This paper mainly focuses on developing and evaluating the ability of the SARIMA model of predicting wastewater inflow rate to the Murray Bridge wastewater network/WWTP in South Australia. The SARIMA method was applied due to its capability to handle shortcoming of the ARIMA model in dealing with seasonal components in the time series. There has been no evidence of this model application in wastewater inflow rate prediction. Besides, low temporal resolution forecast of 60 min for wastewater inflow using ARIMA family models has not been demonstrated in the literature. This paper came to fill these gaps of knowledge.

SARIMA technique was successful in wastewater inflow modelling and forecasting for the case study WWTP at low temporal resolution with hourly time series data. SARIMA (1,0,3)(2,1,2)_24_ was identified as the best model amongst potential ones. The orders (p,d,q) and (P,D,Q) of the proposed SARIMA model were diagnostically checked by performing visualization (ACF and PACF graphs), and statistical test (Ljung-Box test) for the residuals. Short-term forecasts for 1 week ahead were shown for the first 7 days of July 2018. The results indicate the proposed SARIMA model provides high accuracy forecasts based on several evaluation criteria including RMSE, MAPE and *R*^2^.

The wastewater forecasts for low temporal resolution of 60 min generated from the proposed SARIMA model can be utilized as an input for wastewater pump operations optimization model or pump controller in real-time. Wastewater inflow predictions are an important factor in optimizing the pump operations. With high accuracy forecasts, the pumping system reliability is improved, and pump schedules can be set up appropriately in advance with consideration of the predictions of electricity spot prices to obtain electrical energy cost savings.

An advantage of the SARIMA technique is it only requires historical observations to develop forecasting models, as it relies on the behaviour of past data points to predict future data points. However, it is also a limitation as SARIMA could not include other attributes that have influences on the wastewater inflow rate (e.g. rainfall) as its inputs. These influencing factors should be considered for future research to improve the accuracy of the SARIMA wastewater inflow forecasting model. Moreover, a comparative study on forecasting wastewater inflow rate using SARIMA model and machine-learning-based techniques such as artificial neural network (ANN), random forest (RF) and k-nearest neighbour (k-NN) is recommended to be conducted to further evaluate the ability of the SARIMA. An additional possible research direction is to further validate the proposed forecasting model for the Murray Bridge WWTP in this study by comparing its performance against that of SARIMA models developed for other WWTPs. The performing ability of each model for each WWTP case study will be assessed and compared based on a number of statistical criteria (e.g. RMSE, MAPE and *R*^2^). Hourly wastewater inflow datasets of the same length of time period should be utilized to generate low temporal resolution forecasting models for those WWTPs to achieve the most accurate results of comparison. Finally, different modelling and forecasting with lower/higher temporal resolutions such as 15 min, 30 min, daily and monthly should be investigated to support the wastewater pumping system and WWTP for different operation purposes.

## Data Availability

The datasets used and/or analysed during the current study are available from the corresponding author on reasonable request.
